# The 4′-Hydroxyl Group of Resveratrol Is Functionally Important for Direct Activation of PPARα

**DOI:** 10.1371/journal.pone.0120865

**Published:** 2015-03-23

**Authors:** Yoshie Takizawa, Rieko Nakata, Kiyoshi Fukuhara, Hiroshi Yamashita, Hideo Kubodera, Hiroyasu Inoue

**Affiliations:** 1 Department of Food Science & Nutrition, Nara Women’s University, Nara, Japan; 2 Showa University School of Pharmacy, Tokyo, Japan; 3 Medicinal Chemistry Research Laboratories, Mitsubishi Tanabe Pharma Corporation, Kanagawa, Japan; Virginia Commonwealth University, UNITED STATES

## Abstract

Long-term moderate consumption of red wine is associated with a reduced risk of developing lifestyle-related diseases such as cardiovascular disease and cancer. Therefore, resveratrol, a constituent of grapes and various other plants, has attracted substantial interest. This study focused on one molecular target of resveratrol, the peroxisome proliferator activated receptor α (PPARα). Our previous study in mice showed that resveratrol-mediated protection of the brain against stroke requires activation of PPARα; however, the molecular mechanisms involved in this process remain unknown. Here, we evaluated the chemical basis of the resveratrol-mediated activation of PPARα by performing a docking mode simulation and examining the structure-activity relationships of various polyphenols. The results of experiments using the crystal structure of the PPARα ligand-binding domain and an analysis of the activation of PPARα by a resveratrol analog 4-phenylazophenol (4-PAP) *in vivo* indicate that the 4′-hydroxyl group of resveratrol is critical for the direct activation of PPARα. Activation of PPARα by 5 μM resveratrol was enhanced by rolipram, an inhibitor of phosphodiesterase (PDE) and forskolin, an activator of adenylate cyclase. We also found that resveratrol has a higher PDE inhibitory activity (IC_50_ = 19 μM) than resveratrol analogs trans-4-hydroxystilbene and 4-PAP (IC_50_ = 27-28 μM), both of which has only 4′-hydroxyl group, indicating that this 4′-hydroxyl group of resveratrol is not sufficient for the inhibition of PDE. This result is consistent with that 10 μM resveratrol has a higher agonistic activity of PPARα than these analogs, suggesting that there is a feedforward activation loop of PPARα by resveratrol, which may be involved in the long-term effects of resveratrol *in vivo*.

## Introduction

The phytoalexin resveratrol (3, 5, 4′-trihydroxystilbene) [1] possesses antioxidant properties and has multiple effects, including the inhibition or suppression of cyclooxygenase (COX) [2], [3], and the activation of peroxisome proliferator activated receptors (PPARs) [4] and the NAD^+^-dependent protein deacetylase sirtuin 1 (SIRT1) [5]. Previous studies show that resveratrol can prevent or slow the progression of various cancers, cardiovascular diseases, and ischemic injuries, as well as enhancing stress resistance and extending lifespan [6], [7].

Resveratrol is a calorie-restriction mimetic [8] with potential anti-aging and anti-diabetogenic properties; therefore, its ability to activate SIRT1 has attracted particular interest. However, the activation of SIRT1 by resveratrol *in vitro* appears to be an artifact generated by the use of fluorophore-tagged substrates [9], [10]. A recent study reported that cAMP-dependent phosphodiesterase (PDE) is a direct target of resveratrol and suggested that the metabolic effects of the compound are mediated by PDE inhibition [11]; however, this proposal remains unconfirmed. Previous studies by our group focused on the hypothesis that the beneficial effects of resveratrol require the direct activation of PPARα [4], [12], [13], which is supported by reports that PPARα mediates some of the effects of calorie restriction [14].

PPARs are members of a nuclear receptor family of ligand-dependent transcription factors [15]. The three PPAR isoforms, PPARα (NR1C1), β/δ (NR1C2), and γ (NR1C3), show distinct tissue distributions and play various roles in lipid and carbohydrate metabolism, cell proliferation and differentiation, and inflammation, and are considered molecular targets for the treatment of lifestyle-related diseases [15], [16]. The ligand-binding domains of the PPAR isoforms share 60–70% sequence identity, although all three isoforms bind naturally occurring fatty acids [17]. The prostaglandin D_2_-derived metabolite, 15-deoxy-Δ^12, 14^- prostaglandin J_2_, is a potent natural ligand of PPARγ [18], [19]. We previously reported that this metabolite suppresses lipopolysaccharide-induced expression of COX-2, a key inflammatory enzyme in prostaglandin synthesis, in macrophage-like U937 cells but not in vascular endothelial cells [20]. We also demonstrated that the expression of COX-2 is regulated by negative feedback mediated by PPARγ, especially in macrophages [20]. These findings indicate that PPARs participate in the cell type-specific control of COX-2 expression [3], which led us to hypothesize that resveratrol is a direct activator of PPARs. This proposal is supported by the results of *in vitro* reporter assays in bovine arterial endothelial cells (BAECs) [21], which demonstrated that 5 μM resveratrol activates PPARα, β/δ, and γ [4], [13]. In a study using PPARα-knockout mice, resveratrol treatment (20 mg/kg weight/day for 3 days) protected the brain against ischemic injury through a PPARα-dependent mechanism, indicating that resveratrol activates PPARα *in vivo* [4]. Moreover, we also demonstrated that the resveratrol tetramer, vaticanol C, activates PPARα and PPARβ/δ both *in vitro* (5 μM) and *in vivo* (0.04% of the diet for 8 weeks), although no effects on SIRT1 were observed [13].

In light of the findings described above, the aim of this study was to evaluate the chemical basis of the activation of PPARα by resveratrol.

## Materials and Methods

### Reagents and cell culture

Resveratrol was purchased from Sigma and the other plant polyphenols were purchased from Wako Chemicals (Japan). Azobenzene and 4-phenylazophenol (4-PAP) were purchased from Tokyo Chemicals, and trans-4-hydroxystilbene (T4HS) was synthesized as reported previously [22]. A 100 mM stock solution of each compound was prepared in DMSO and the stock was diluted to the working concentration before use. BAECs (Cell Applications, San Diego, CA) were grown in DMEM supplemented with 10% fetal calf serum.

### Transcription assays and construction of mutated PPARα expression vectors

BAECs were transfected with 0.15 μg of the tk-PPREx3-Luc reporter plasmid, 0.15 μg of the human PPARα expression vector pGS-hPPARα (GeneStorm clone L02932; Invitrogen), and 0.04 μg of the pSV-βgal vector, using Trans IT-LT-1 (Mirus) as described previously [20], [23]. Twenty-eight hours after transfection, the BAECs were incubated with the relevant chemical for 24 h, after which the cells were harvested and lysed, and luciferase and β-galactosidase activities were measured. The luciferase activities were normalized to those of the β-galactosidase standard. The validity of this reporter assay was previously confirmed using Wy-14643, GW501516, and pioglitazone, which are synthetic agonists of PPARα, β/δ, and γ, respectively [23]. Site-directed mutagenesis of PPARα to form I241A, L247A, F273A, I317A and I354A was performed using an inverse PCR method, the KOD-Plus-Mutagenesis Kit (Toyobo, Japan), pGS-hPPARα as a template, and mutagenic primers. Mutagenic primers used were: F273A 5′- gctcactgctgccagtgcacgtcagtggagaccgtcac-3′ (forword), 5′- gatgcggacctccgccaccaagttcaggatgccattgg-3′ (reverse); I354A 5′- gccatggaacccaagtttgattttgc catgaagttcaat-3′ (forword), 5′- atcacagaacggtttccttaggctttttaggaattcacg-3′ (reverse); I241A 5′- gcacatgatatggagacactgtgtatg-3′ (forword), 5′- tgcgacaaaaggtggattgttactg-3′ (reverse); L247A 5′- agcatgtatggctgagaagacgctgg-3′ (forword), 5′- gccatacatgctgtctccatatcatgtatgac-3′ (reverse); I317A 5′- gcattcgccatgctgtcttctgtg-3′ (forword), 5′- tgcggcctcataaactccgtattttagc-3′ (reverse). All mutations were confirmed by DNA sequencing.

### Docking mode prediction and free energy calculations

The docking modes of resveratrol were predicted using the GOLD 3.0 docking program [24]. The protein co-ordinates were taken from the PPARα-GW409544 complex structure (PDB ID: 1K7L) and the amino acid residues within 12 Å of GW409544 were assumed to be the target binding site. The docking procedure with GOLD 3.0 was repeated 150 times, and the 150 docking poses were clustered to obtain four representative poses. Molecular dynamics simulations were performed using the AMBER 8 program and the Cornell force field 94. The solvent water was the SPC model and the cubic periodic boundary condition was used. The Coulomb interaction was evaluated using the particle mesh Ewalt method. The protein-ligand complex structure was moved with a time step of 2 femtoseconds and hydrogens were constrained with the SHAKE algorithm. After standard minimization and equilibration of the protein-ligand complex, simulation was performed for 1 nanosecond and 1,000 snapshots were collected. A Molecular Mechanical/Poisson-Boltzmann Surface Area analysis [25] was performed with a standard protocol. Computational alanine scanning was performed in a similar manner to that described above, mutating each amino acid in turn.

### Animal experiments

Male 8-week-old SV/129-strain (wild-type) and PPARα-knockout mice (Jackson Laboratory) were housed in a room at 24 ± 2°C with a 12 h/12 h light/dark cycle and were fed the AIN93-G diet or the same diet supplemented with 0.04% 4-PAP. Food and water were available ad libitum. After 8 weeks of feeding, the mice were anesthetized with isoflurane, and euthanized by collecting a blood sample using a syringe. Livers were removed and stored in RNA later solution (Ambion, USA) at -30°C. Body weight, food consumption and liver weight were not significantly different between 4-PAP-fed mice and control. In addition, plasma AST and ALT level of 4-PAP-fed mice were same level as control (data not shown). This study was carried out in accordance with the guideline for Care and Use of Laboratory Animals published by Minister of the Environment Government of Japan (No. 88 of April 28, 2006). All experimental procedures were approved by the Animal Care Committee of Nara Women’s University. All efforts were made to minimize suffering.

### Real-time PCR

Total RNA was isolated using the acid guanidinium thiocyanate procedure. Real-time RT-PCR was performed using the Mx3005 system (Stratagene) as described previously [23]. Expression levels of each mRNA were normalized to those of GAPDH mRNA. PCR primers used were: GAPDH 5′- ggtgaaggtcggagtcaacgga-3′ (forword), 5′- gagggatctcgctcctggaaga-3′ (reverse); Acyl CoA oxidase 1 5′- gggagtgctacgggttacatg-3′ (forword), 5′- ccgatatccccaacagtgatg-3′ (reverse); Carnitine palmitoyltransferase 1A 5′- cttccatgactcggctcttc-3′ (forword), 5′- aaacagttccacctgctgct-3′ (reverse); Adiponectin receptor type 2 5′- acccacaaccttgcttcatc-3′ (forword), 5′- ggcagctccggtgatataga-3′ (reverse); Fatty acid binding protein 1 5′- aagtaccaattgcagagccagga-3′ (forword), 5′- ggtgaactcattgcggacca-3′ (reverse); Long-chain acyl CoA dehydrogenase 5′- cagttgcatgaaaccaaacg-3′ (forword), 5′- gacgatctgtcttgcgatca-3′ (reverse); SIRT1 5′- gtcagataaggaaggaaaac-3′ (forword), 5′- tggctctatgaaactgttct-3′ (reverse).

### PDE inhibition assay

The PDE inhibition assay was performed using the PDE-Glo^TM^ Phosphodiesterase assay (Promega). Bovine brain-derived PDE, majority of which was PDE4 isozyme [26], was purchased from Sigma. One milliunit of PDE was pre-incubated with varying concentrations of rolipram (Wako Chemicals, Japan), resveratrol, T4HS or 4-PAP for 30 min at room temperature, and then 1 μM cAMP substrate was added and the reactions were incubated for a further 90 minutes at 37°C. Luminescence was measured using the Tecan Infinite 200 plate-reader.

### Statistical analysis

All results are expressed as the mean ± SD. Comparisons between groups were performed using unpaired *t*-tests or two-way ANOVA with post-hoc Bonferroni multiple comparison test. Values were deemed to be statistically significantly different at *p* < 0.05.

## Results and Discussion

### The 4′-hydroxyl group of resveratrol is required for the activation of PPARα *in vitro*

**Fig 1:**
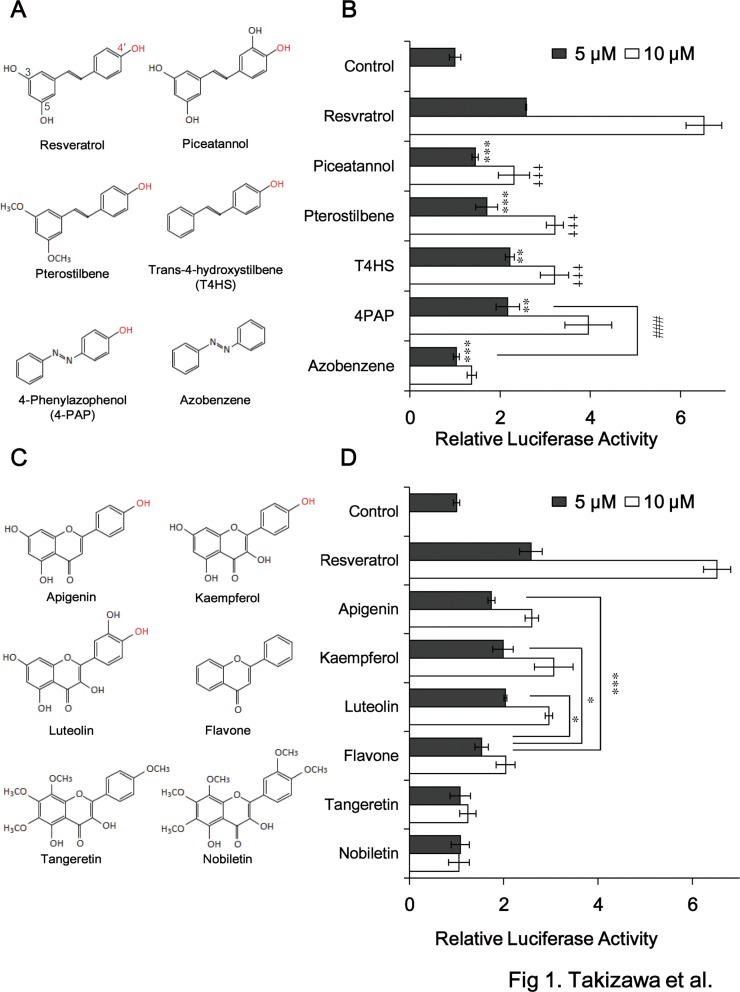
The 4′-hydroxyl group of resveratrol is required for the activation of PPARα *in vitro*. (A) The chemical structures of resveratrol and its related compounds containing a 4′-hydroxyl group (shown in red). (B) The activation of PPARα by exposure of BAECs transiently transfected with PPRE-luc, GS-hPPARα, and pSV-β-gal to the compounds (5, 10 μM) shown in (A). Data were statistically evaluated using the unpaired *t*-test. ** *p* < 0.01, ***p < 0.001 compared with cells treated with 5 μM resveratrol. †††*p* < 0.001 compared with cells treated with 10 μM resveratrol. ^###^ *p* < 0.001 compared with cells treated with 4-PAP. (C) The chemical structures of the flavonoids studied. (D) The activation of PPARα by exposure of BAECs transiently transfected with PPRE-luc, GS-hPPARα, and pSV-β-gal to 5 μM of resveratrol or to 5 μM of the flavonoids shown in (C). Data were statistically evaluated using the unpaired *t*-test. *p < 0.05, ***p < 0.001 compared with cells treated with flavone. (B) and (D) were presented as the relative luciferase activities normalized to those of the β-galactosidase standard, and represent the mean ± SD of three independent wells of cells. Similar results were obtained by two additional experiments.

First, we investigated whether resveratrol and its related compounds (Fig. 1A) are able to activate PPARα in a cell-based luciferase reporter assay. BAECs were transiently transfected with the PPRE-luc reporter vector, the human PPARα expression vector GS-hPPARα, and pSV-β-gal as an internal control, and then incubated with 5, 10 μM resveratrol or its related compounds for 24 h. The activation of PPARα by resveratrol was suppressed by the addition of a 3′-hydroxyl group (to form piceatannol), by the replacement of the 3,5-hydroxyl groups with methoxy groups (to form pterostilbene), and by deletion of the 3,4-hydroxyl groups from resveratrol (to form T4HS) (Fig. 1A, B). The activation of PPARα by 4-PAP, which has a chemical structure similar to that of T4HS instead of the stilbene to azobenzene backbone (Fig. 1A), was similar to that by T4HS, and that, the level of activation was reduced further following deletion of the hydroxyl group (to form azobenzene) These compounds showed the dose-dependent increase of PPARα activation except for azobenzene (Fig. 1A, B).

Next, we compared the PPARα-activating ability of other polyphenols with a flavone backbone (Fig. 1C) with that of resveratrol. The compounds studied were as follows: apigenin, which has a similar 4′-hydroxyl group to that of resveratrol; kaempferol and luteolin, both of which have chemical structures similar to that of apigenin but contain an additional one or two hydroxyl groups, respectively; a flavone with no hydroxyl group; and tangeretin and nobiletin, which have four or five methoxy groups, respectively, one of which replaces the 4′-hydroxyl group of resveratrol (Fig. 1C). The abilities of apigenin, kaempferol, and luteolin to activate PPARα were approximately 20–35% lower than that of resveratrol (Fig. 1D). The flavone that lacked hydroxyl groups displayed 55% of the activating ability of resveratrol and the ability of flavone to activate PPARα was significantly lower than that of apigenin, kaempferol and luteolin. The abilities of these compounds to activate PPARα at 10 μM were higher than 5 μM except for tangeretin and nobiletin (chemicals with no “corresponding 4’-OH”). These results indicate that the 4′-hydroxyl group of resveratrol is functionally important for the activation of PPARα although the contribution of this 4′-hydroxyl group may differ between the stilbene and flavone backbones.

### Identification of a plausible docking model and identification of F273 and I354 as PPARα residues involved in resveratrol binding

**Fig 2:**
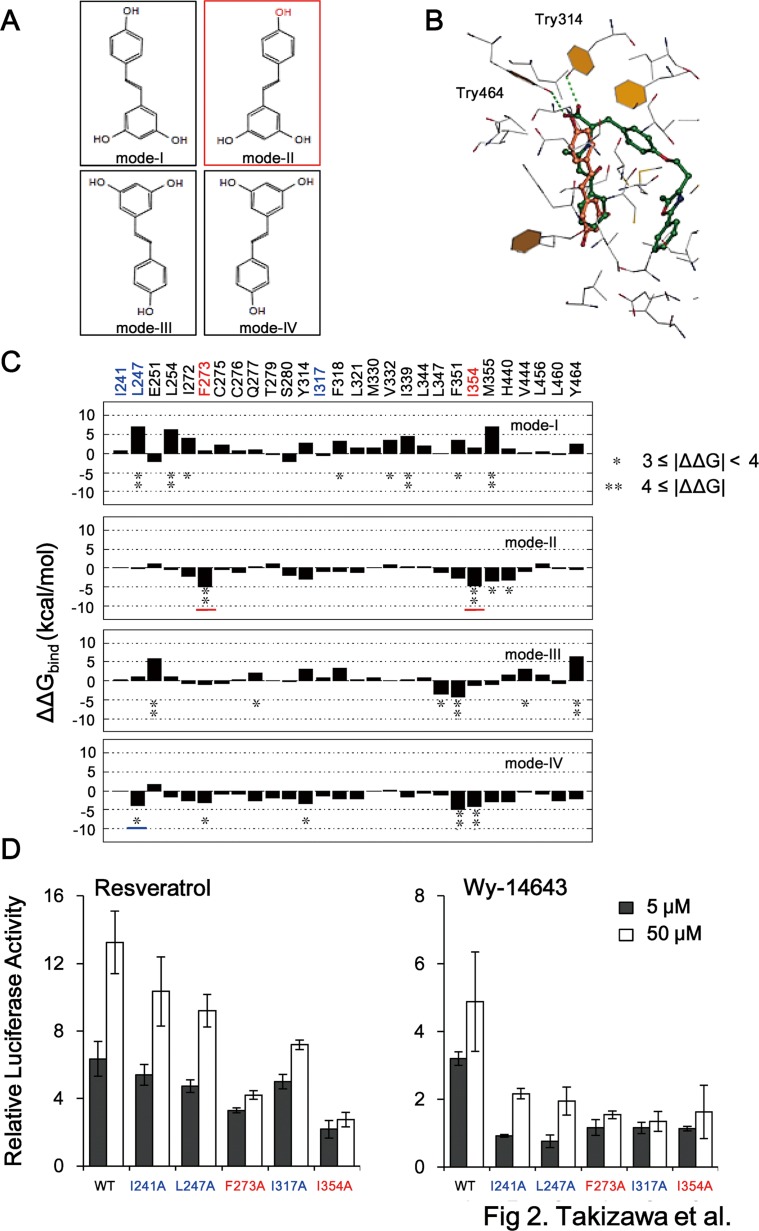
Docking models and analysis of PPARα residues required for binding to resveratrol. (A) The four docking modes of resveratrol predicted using the GOLD 3.0 docking program [24] with protein co-ordination data from the PPARα-GW409544 complex structure (PDB ID: 1K7L) and a standard docking protocol. (B) Superimposition of docking mode II of resveratrol (orange) on the structure of PPARα bound to GW409544, a potent PPARα agonist (green). Only the amino acids located near to GW409544 are displayed. The hydrogen bonds of Tyr314 and Tyr464 are shown as dashed green lines. (C) Binding free energies (∆∆Gbind (kcal/mol)) of the indicated PPARα amino acid residues, calculated by alanine scanning using data for the four predicted docking modes. (D) Activation of wild-type (WT) PPARα and its mutants by 5, 50 μM resveratrol or Wy-14643. BAECs were transiently transfected with PPRE-luc, wild-type or mutant GS-hPPARα, and pSV-β-gal. The data are presented as relative luciferase activities normalized to those of the β-galactosidase standard and as 1 for cells treated with DMSO (control), and represent the mean ± SD of three independent wells of cells. Similar results were obtained by two additional experiments. The data were calculated the relative luciferase activity in cells transfected with wild-type PPARα.

The X-ray crystal structure of the PPARα LBD as a complex with its synthetic agonist GW409544 and a co-activator motif from steroid receptor co-activator 1 was reported previously [27]. The hydrogen bonds between the carboxylate of GW409544, Tyr314 on helix 5, and Tyr464 on the AF2 helix, act as a molecular switch that activates the transcriptional activity of PPARα [27]. The docking modes of resveratrol were predicted using the GOLD 3.0 docking program [24] and protein co-ordinates from the PPARα-GW409544 complex structure (PDB ID: 1K7L). Four modes were predicted; the four orientations of the nearly planar molecule are horizontal or vertical mirror images (Fig. 2A). Of the four predicted modes, modes I and II, which are vertical mirror images, seem feasible for two reasons. First, when the calculated docking mode II of resveratrol was superimposed on the PPARα-GW409544 complex structure, the configuration of resveratrol (Fig. 2B; orange) partially overlapped that of GW409544 (Fig. 2B; green). Second, the 4′-hydroxyl group of resveratrol was in the vicinity of the hydroxyl groups of Tyr314 and Tyr464, suggesting the possibility of hydrogen bond formation between them. The 3,5-hydroxyl groups of resveratrol were located near to hydrophobic amino acid residues, suggesting that they do not contribute much to the binding affinity for PPARα. This proposal is consistent with the finding that removing these groups (to form T4HS) had a slight but significant suppressive effect on the ability of resveratrol to activate PPARα (Fig. 1B). The binding features were also consistent with the experimental observation that the 4′-hydroxyl group is a crucial functional moiety for PPARα activation (Fig. 1).

In modes III and IV, which are horizontal mirror images of modes II and I, respectively, the 4′-hydroxyl group would be located further away from Tyr314 and Tyr464; therefore, these modes may not be compatible with the apparent importance of this group to PPARα activation. However, the binding free energies predicted using a Molecular Mechanical/Poisson-Boltzmann Surface Area analysis [25] showed that modes II (-10.28 ± 9.12 kcal/mol) and IV (-15.64 ± 9.31 kcal/mol) are more plausible than mode I (-1.28 ± 11.12 kcal/mol), although it is worth noting that the free energy for GW409544 binding is-35.63 ± 11.79 kcal/mol. Ideally, these calculations should be based on crystallographically determined complex co-ordinates, although we resorted to docking predictions here. Taken together, this information suggests that mode II is the most plausible docking model for resveratrol (Fig. 2C).

A computational alanine scanning technique was then used to examine the contribution of each PPARα amino acid residue around the ligand. We were predicted that the residues F273 and I354 were the most favorable sites for binding the free energy of resveratrol in mode II whereas the residues I241, L247 and I317 were not favorable sites in mode II. Consistent with these predictions, site-directed mutagenesis of either of these residues (F273A or I354A) reduced the activation of PPARα by resveratrol compared with others (I241A, L247A, and I317A) (Fig. 2D) in BAECs transiently transfected with the PPRE-luc reporter. On the other hand, all mutants (I241A, L247A, F273A, I317A, and I354A) were suppressed by Wy-14643. These results provide additional evidence that docking mode II of resveratrol is plausible, and that its 4′-hydroxyl group is functionally important for PPARα activation. In this study, we did not show that resveratrol directly binds to PPARα, however, our collaborated study showed the direct interaction between resveratrol and PPARγ by X-ray crystal structure analysis (unpublished data), which is also recently reported by another group [28].

### 4-PAP induces the expression of PPARα-dependent genes and SIRT1

**Fig 3:**
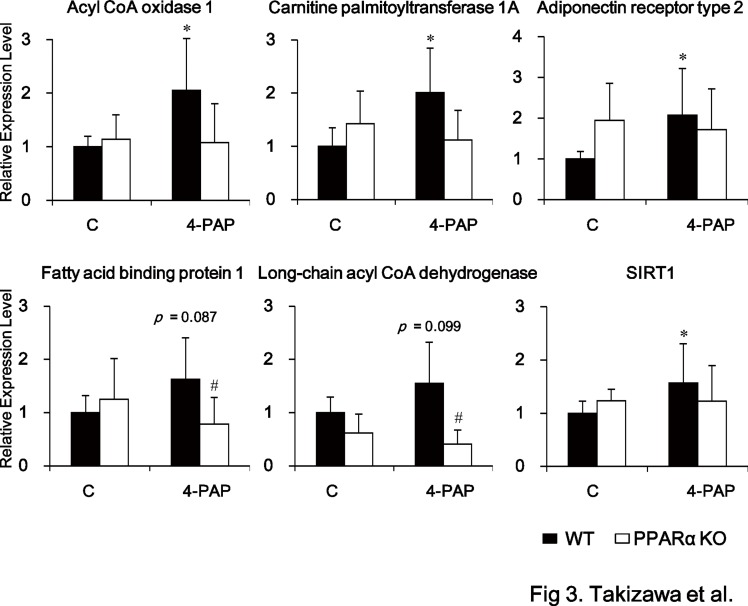
4-PAP induces PPARα-dependent genes and SIRT1 *in vivo*. RT-qPCR was used to determine the mRNA levels of the indicated genes in liver samples from wild-type (WT; filled columns) and PPARα-knockout (PPARα KO; open columns) mice fed the control AIN-93G diet (C) or the same diet supplemented with 0.04% 4-PAP for 8 weeks. Data represent the mean ± SD from 7–8 mice in each group (WT) and from 4 mice in each group (PPARα KO). Data were statistically evaluated using the unpaired two-way ANOVA with post-hoc Bonferroni multiple comparison test. *p < 0.05 compared with wild-type mice fed the control diet. ^#^ *p* < 0.05 compared with wild-type mice fed the 4-PAP-supplemented diet. For each mRNA, data were normalized to the expression levels in wild-type mice fed the control diet.

Next, the importance of the 4′-hydroxyl group of resveratrol to the activation of PPARα *in vivo* was examined. A previous study demonstrated that exposure of wild-type mice to 0.04% vaticanol C, a resveratrol tetramer, upregulates the hepatic expression of PPARα-responsive genes such as fatty acid binding protein 1. However, this response was not observed in PPARα-knockout mice, indicating that vaticanol C activates PPARα *in vivo* [13]. Similarly, we recently found that exposure of wild-type mice (but not PPARα-knockout mice) to 0.04% resveratrol for 4 weeks upregulates the hepatic expression of SIRT1 and PPAR-responsive genes such as Acyl CoA oxidase 1, Long-chain acyl CoA dehydrogenase, and Fatty acid binding protein 1 (unpublished data), indicating that resveratrol also activates PPARα *in vivo*. Here, a resveratrol analog 4-PAP, which has a 4′-hydroxyl group on an azobenzene backbone (Fig. 1A), was used to examine the importance of this group to the activation of PPARα *in vivo*. Compared with wild-type mice fed a control diet, those exposed to 0.04% 4-PAP for 8 weeks showed significantly higher hepatic expression levels of the PPARα-responsive genes such as Acyl CoA oxidase 1, Carnitine palmitoyltransferase 1A and Adiponectin receptor type 2 and a tendency toward higher expression levels of the genes such as Fatty acid binding protein 1 and Long-chain acyl CoA dehydrogenase (Fig. 3). These responses were not observed in PPARα knockout mice, indicating that 4-PAP activates PPARα *in vivo* (Fig. 3). Interestingly, similar to the results of our experiments using resveratrol (unpublished data), there was significantly 4-PAP-induced upregulation of SIRT1mRNA expression in wild-type, but not PPARα knockout mice (Fig. 3), indicating that PPARα-dependent upregulation of SIRT1 mRNA is attributable to SIRT1-activation by resveratrol *in vivo*.

### Inhibition of PDE enhances the activation of PPARα by resveratrol

**Fig 4:**
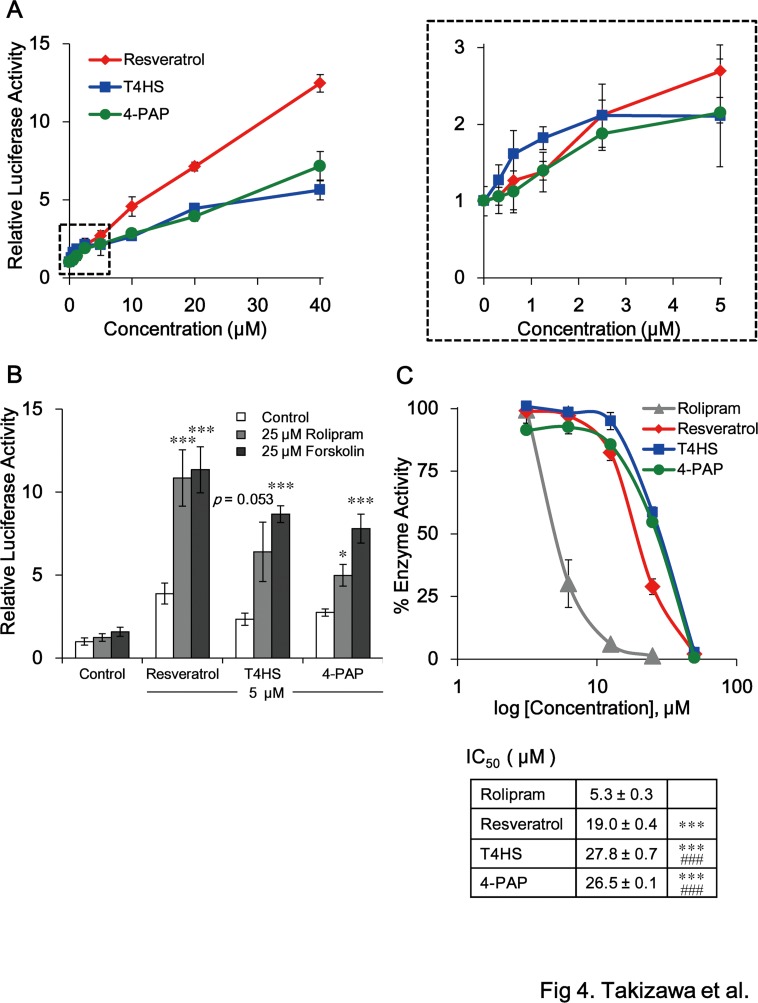
Inhibition of PDE enhances the activation of PPARα by resveratrol, especially at higher doses. (A) The dose-dependent activation of PPARα by resveratrol, T4HS and 4-PAP in BAECs transiently transfected with PPRE-luc, GS-hPPARα, and pSV-β-gal. Following transfection, the cells were incubated for 24 h with resveratrol, T4HS or 4-PAP at the indicated concentrations. Data were normalized to the β-galactosidase standard and represent the mean ± SD of three independent wells of cells. The right graph corresponds to the lower area marked by a dashed rectangle in left graph. (B) cAMP-dependent enhancement of PPARα activation by resveratrol, T4HS or 4-PAP. BAECs transiently transfected with PPRE-luc, GS-hPPARα, and pSV-β-gal were incubated for 24 h with 5 μM compounds in the presence or absence of 25 μM rolipram, a PDE4 inhibitor, or 25 μM forskolin, an adenylate cyclase activator. Luciferase data were normalized to the β-galactosidase standard and represent the mean ± SD of three independent wells. *p < 0.05, ***p < 0.001 (unpaired *t*-test) compared with control cells treated with the same compound. (C) The inhibition of PDE by resveratrol, T4HS, and rolipram. Data represent the mean ± SD of three independent wells of cells. Similar results were obtained by two additional experiments. The IC_50_ values are shown in the Table. ***p < 0.001 (unpaired *t*-test) compared with rolipram. ^###^ *p* < 0.001 (unpaired *t*-test) compared with resveratrol. Similar results were obtained by two additional experiments in (A-C).

Finally, the inhibitory effect of PDEs on the activation of PPARα by resveratrol was examined using a luciferase reporter assay. BAECs were transiently transfected with the PPRE-luc reporter vector, the human PPARα expression vector GS-hPPARα, and pSV-β-gal as an internal control, and then incubated with varying concentrations of resveratrol, T4HS or 4-PAP for 24 h. At higher concentrations (from 10 μM to 40 μM), resveratrol had a more potent effect on the activation of PPARα than the others (Fig. 4A, left), on the other hand, resveratrol, T4HS and 4-PAP had the similar effect at lower concentrations (from 1.25 to 2.5 μM) (Fig. 4A, right). These results suggest that the 4′-hydroxyl group of resveratrol contributes to the activation of PPARα at up to 2.5 μM concentration, however, this 4′-hydroxyl group is not sufficient for the PPARα-activation at over 10 μM concentration.

A recent study reported that resveratrol inhibits PDE isozymes, PDE3 (IC_50_ = 10 μM) and PDE4 (IC_50_ = 14 μM), respectively [11]. It is therefore possible that the more potent effect of higher concentrations of resveratrol on the activation of PPARα is dependent on the inhibition of PDE, which will be contributed to the subsequent increase in intracellular cAMP levels. The activation of PPARα by 5 μM resveratrol, T4HS, or 4-PAP was enhanced by rolipram, a PDE4 inhibitor, or forskolin, an adenylate cyclase activator, both of which increase intracellular cAMP levels, although rolipram or forskolin alone could not activate PPARα (Fig. 4B). These results indicate that the activation of PPARα by resveratrol or its related compound is enhanced by cAMP. Thus, PPARα activation by resveratrol at an early point serves as a trigger to enhance the activation of PPARα in advance of the inhibition of PDE by resveratrol. Our PDE inhibition assay (Fig. 4C) revealed that resveratrol is a more potent inhibitor (IC_50_ = 19.0 μM) than T4HS (IC_50_ = 27.8 μM; *p* = 0.00012) and 4-PAP (IC_50_ = 26.5 μM; *p* = 0.00022), which explains the relatively greater effect of higher 10 μM concentration of resveratrol on the activation of PPARα (Fig. 4A). Zhao *et al*. recently reported that by different PDE4 assay using ^3^H-cAMP, resveratrol is more potent inhibitor (IC_50_ = 14.0 μM) than pterostilbene (Fig. 1A) (IC_50_ = 27.0 μM) [29], which is similar to our PDE inhibitory data of T4HS and 4-PAP (Fig. 4C), indicating that the 4′-hydroxyl group of resveratrol partly contributes, but not sufficient, to inhibition of PDE.

This study investigated the molecular mechanisms involved in the activation of PPARα by resveratrol. An examination of the structure-activity relationships of resveratrol-related compounds revealed that the 4′-hydroxyl group of resveratrol is functionally important for the direct activation of PPARα (Fig. 1). This result was confirmed by a docking model simulation and a subsequent experiment using the crystal structure of the PPARα LBD (Fig. 2), as well as by an *in vivo* investigation of PPARα activation by resveratrol analog 4-PAP (Fig. 3). Remarkably, the induction of SIRT1 mRNA depends on the activation of PPARα by 4-PAP (Fig. 3) and resveratrol (unpublished data). Although direct activation of SIRT1 by resveratrol was unclear [9], [10], SIRT1 was reported to bind to PPARα and enhanced the transcriptional activity of PPARα with its co-activator PGC-1α and promotes fatty acid oxidation [30]. Therefore, there may be a feedforward activation of PPARα by resveratrol via activation of SIRT1.

**Fig 5:**
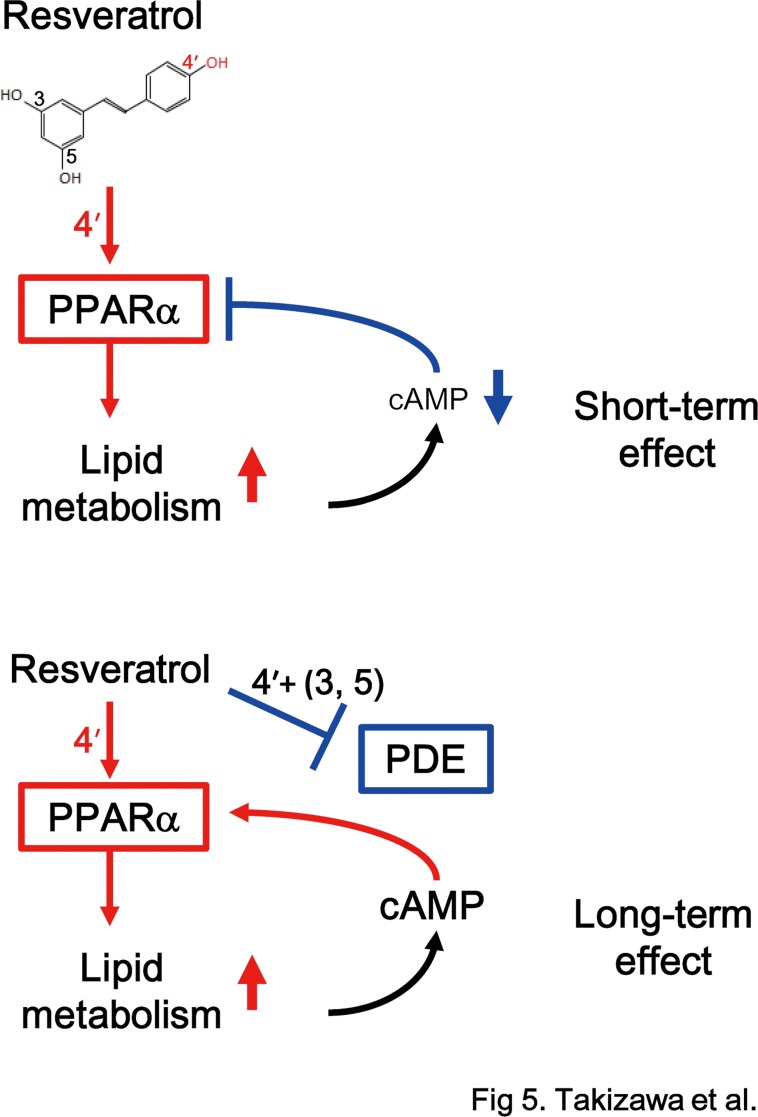
Possible relationship among resveratrol, PPARα and PDE. These diagrams present our hypothesis about short- and long-term effects of resveratrol, as shown in the text.

Whereas the 4′-hydroxyl group of resveratrol directly contributes to PPARα activation, this 4′-hydroxyl group partly contributes to inhibition of PDE since the pattern of inhibition differed between resveratrol, T4HS and 4-PAP (Fig. 4). Activation of PPARα by resveratrol was enhanced by its inhibition of PDE. This feedforward activation of PPARα by resveratrol may provide a reasonable explanation why long-term intake of resveratrol at concentrations lower than those used for *in vitro* assays induces the activation of PPARα *in vivo*. Fig. 5 shows an ongoing hypothesis on long-term activation of PPARα by resveratrol *in vivo*. As a short-term effect, resveratrol activates PPARα, which induces PPARα responsive genes involved in lipid metabolism. Activation of lipid metabolism finally increases intracellular ratio of ATP/ADP, and will decrease intracellular cAMP levels, which may feedback control of PPARα–activation with a time lag. As a long-term effect, resveratrol inhibits PDE, which will enhance the PPARα-activation. At present, we do not have sufficient evidences for this hypothesis, especially feedback regulation of PPARα *in vivo*. Further study will need to evaluate this hypothesis.
